# Tyrosine kinases in nodal peripheral T-cell lymphomas

**DOI:** 10.3389/fonc.2023.1099943

**Published:** 2023-02-08

**Authors:** Pier Paolo Piccaluga, Chiara Cascianelli, Giorgio Inghirami

**Affiliations:** ^1^ Biobank of Research, IRCCS Azienda Opedaliera-Universitaria di Bologna, Bologna, Italy; ^2^ Department of Experimental, Diagnostic, and Specialty Medicine, School of Medicine, University of Bologna, Bologna, Italy; ^3^ Immunopathology and Hematopathology, Weill Cornell Medical College, New York-Presbyterian Hospital, New York, NY, United States

**Keywords:** peripheral T-cell lymphoma, anaplastic large cell lymphoma, follicular T-cell lymphoma, PDGFRA = PDGFR alpha, JAK/STAT (janus kinase/signal transducer and activator of transcription), tyrosine kinase inhibitors (TKI), ALK (anaplastic lymphoma kinase), ITK/SYK rearrangement

## Abstract

Nodal peripheral T-cell lymphomas (PTCL) are uncommon and heterogeneous tumors characterized by a dismal prognosis. Targeted therapy has been proposed. However, reliable targets are mostly represented by a few surface antigens (e.g., CD52 and CD30), chemokine receptors (e.g., CCR4), and epigenetic gene expression regulation. In the last two decades, however, several studies have supported the idea that tyrosine kinase (TK) deregulation might be relevant for both the pathogenesis and treatment of PTCL. Indeed, they can be expressed or activated as a consequence of their involvement in genetic lesions, such as translocations, or by ligand overexpression. The most striking example is ALK in anaplastic large-cell lymphomas (ALCL). ALK activity is necessary to support cell proliferation and survival, and its inhibition leads to cell death. Notably, STAT3 was found to be the main downstream ALK effector. Other TKs are consistently expressed and active in PTCLs, such as PDGFRA, and members of the T-cell receptor signaling family, such as SYK. Notably, as in the case of ALK, STAT proteins have emerged as key downstream factors for most of the involved TK.

## 1 Introduction

Since the approval of imatinib mesylate (Glivec or Gleevec) for treating Philadelphia chromosome-positive leukemias in 2000 (1), tyrosine kinases have become increasingly attractive therapeutic targets in human cancer (1–4). Therefore, they have been largely investigated in terms of expression and function, and several recurrent mutations have been identified in different cancer types, including solid and hematological malignancies (2, 5).

As far as the latter is concerned, several targets have been identified and characterized, leading to the development and approval of many different tyrosine kinases inhibitors (TKI) (6, 7). The most widely targeted TKs include ABL1, KIT, FLT3, BTK, JAK family members, and PDGFRs (6, 7). Interestingly, lymphomas despite being the most common hematological tumors, have benefitted from TKI, probably less than leukemias, for some years. More recently, BTK inhibitors have become the standard treatment for B-cell malignancies such as mantle cell lymphoma and chronic lymphocytic leukemia (8, 9). In contrast, T-cell lymphomas/leukemias have so far been partially neglected; the only exception represented by anaplastic large cell lymphoma (ALCL) ALK+ (10).

In this article, the Authors review the most recent and relevant data on TK expression in PTCL, focusing on the commonest nodal subtypes, i.e. PTCL/NOS, T-follicular helper (TFH) related PTCLs, and ALCL.

## 2 ALK signaling in anaplastic large cell lymphoma

In the latest edition of the WHO classification, ALCL is divided into four main categories: ALK+, ALK-, primary cutaneous, and breast implant-associated (11). ALK+ ALCL is defined as the presence of genetic rearrangement of the anaplastic lymphoma kinase (ALK) gene. ALK encodes the 210 kDa tyrosine kinase (TK) receptor (CD247), which belongs to the insulin growth factor receptor (IR) superfamily, and its genomic locus is located at the chromosomal band 2p23 (10, 12–14).

Physiologically, ALK is highly expressed in the nervous system during embryogenesis but not in adults (15). Its precise role remains unknown, but some evidence suggests its involvement in neuronal differentiation (10). ALK protein activation is induced by ligands, activation mutations, and fusion proteins, leading to a decrease in apoptosis (16).

In cancer, virtually all genomic breakpoints leading to ALK chimeras are located within the intron between exons 19 and 20 (NM_004304.3), leading to the fusion of the intracytoplasmic domain of ALK (exons 20–29) with different partners, which provide dimerization domains (10, 17, 18).

**Figure 1:**
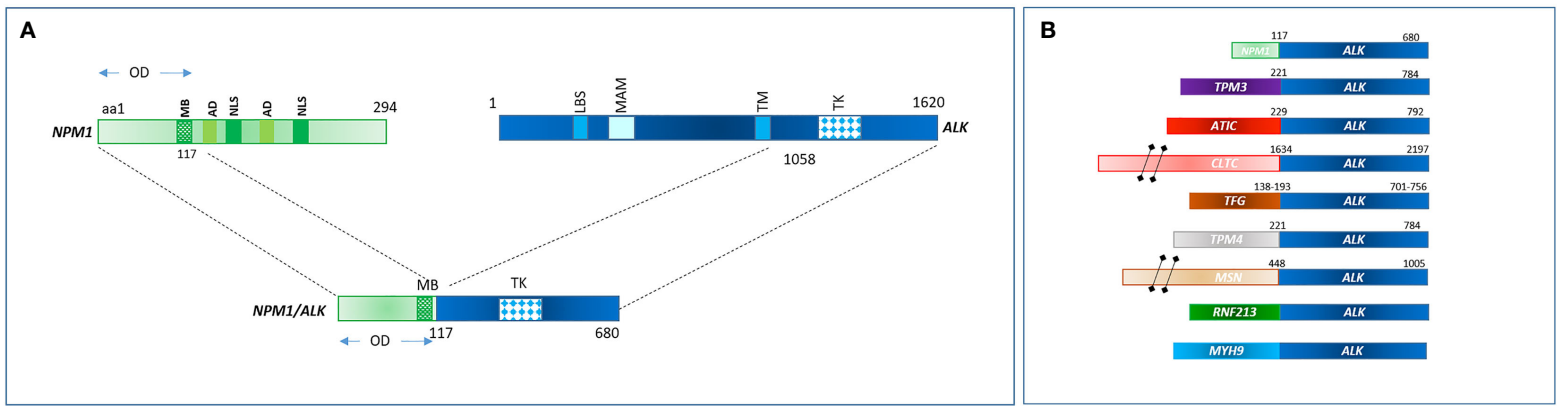
**(A)** *NPM1-ALK* rearrangement in ALCL as consequence of t(2;5)(p23;q35). **(B)** Commonest fusion genes involving ALK in ALCL.

**Figure 2:**
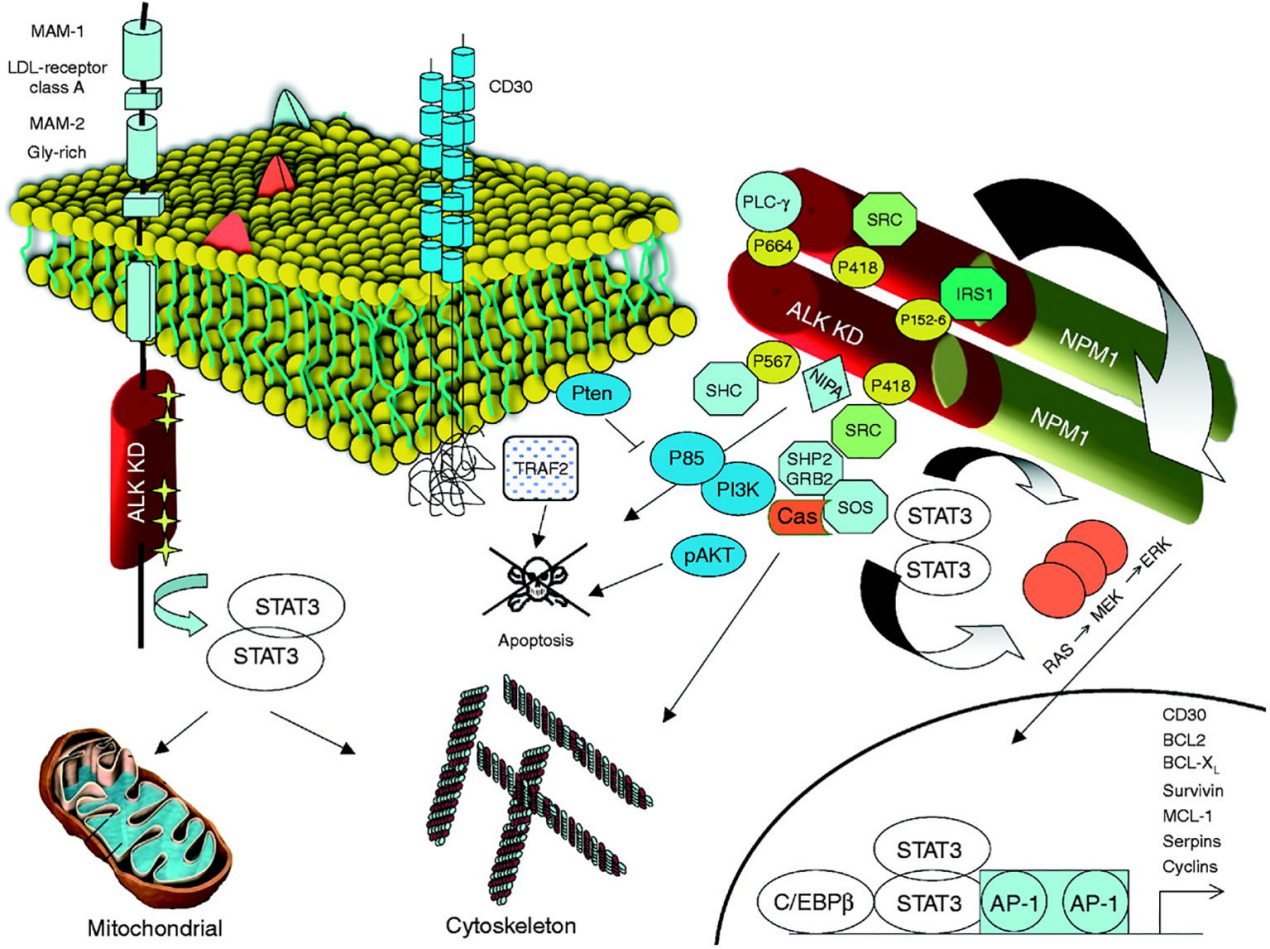
ALK and its signaling transduction pathways. Mutated ALK-R and ALK fusion proteins can elicit multiple signaling pathways, which are responsible for cell transformation and for the maintenance of the neoplastic phenotype. The kinase activation, in both mutated ALK and ALK chimeras, is associated with the docking of several adaptors, which in turn fire several signaling pathways. A critical oncogenic player is represented by the JAK/STAT3 pathway, which provides essential survival signals and modulates the cellular metabolism regulating the mitochondrial oxidation chain. STAT3 is activated by ALK either directly or through JAKs. Reproduced from Journal of Molecular Endocrinology 47, 1; 10.1530/JME-11-0004.

The most common translocation in ALK+ ALCL is t (2, 5)(p23;q25), which causes the expression of an NPM1–ALK fusion protein (**Table 1** and **Figure 1**) (14). NPM1 is a multifunctional protein that acts as a molecular chaperone in the transport of pre-ribosomal particles from the nucleus to the cytoplasm, although it also plays a critical role in DNA repair, transcription, and genomic stability (19). The N-terminal domain of NPM1, within the ALK chimera, provides a dimerization domain essential for autophosphorylation, allowing constitutive activation of the kinase and firing of downstream signaling (17, 20, 21). Functional studies have indicated several NPM1–ALK interacting molecules mediate cellular proliferation (PLC-γ, S-SRC), cell growth (RAS, S-SRC), anti-apoptotic effects (PI3P), and cell migration (S-SRC) (10, 17; **Figure 2**). The oncogenic activity of ALK fusion proteins is largely mediated by STAT3 in ALCL, and STAT3 activation is required for neoplastic phenotype maintenance (22). NPM1–ALK can directly phosphorylate STAT3 or activate JAK3, which in turn can contribute to STAT3 activation (17). STAT3 phosphorylation induces expression of BCL2, BCLXL, survivin, and MCL1 proteins, resulting in anti-apoptotic effects. STAT3-mediated signaling also determines uncontrolled proliferation by interacting with CCND3 and MYC (17).

**Table 1:** Commonest chromosomal translocations involving *ALK* gene in ALCL.

Fusion protein	Chromosomal abnormality	Frequency in ALCL	Cellular localization
NPM–ALK	t(2;5)(p23;q35)	75%	N/C
TPM3–ALK	t(1;2)(q25;p23)	18%	C
ATIC–ALK	inv (2)(p23;q35)	2%	C
CLTC–ALK	t(2;17)(p23;q23)	2%	C
RNF213–ALK	t(2;17)(p23;q25)	1%	C
TFG–ALK	t(2;3)(p23;q21)	1%	C
MSN–ALK	t(2;X)(p23;q11–12)	<1%	CM
TPM4–ALK	t(2;19)(p23;p13)	<1%	C
MYH9–ALK	t(2;22)(p23;q11.2)	<1%	C

Clinically, ALK+ ALCL shows an overall better prognosis than ALK- cases; however, careful attention must be paid to concomitant prognostic factors, such as patient age, since in age-matched series, the difference is not as striking (18).

In contrast, remarkably, ALK is a suitable therapeutic target. Experimentally, in preclinical models, ALK knockdown led to cell cycle arrest, followed by massive apoptosis *in vitro* and/or *in vivo* (23). Similar results were observed for small molecules. Crizotinib (Xalkori), a multitarget TKI targeting ALK, ROS1, and MET, has been approved for treating relapsed refractory ALCL in children and young adults (24–28). Approval was obtained based on a high overall response rate (88%), complete response rate (81%), and duration of response, with 67% of patients not undergoing stem cell transplantation reaching at least 6 months of CR. Cases of long-lasting remission at 28 months and 31 months have also been reported. The effect was relatively rapid, as the median time to the first response was 3.9 weeks (28). Long term results confirmed the efficacy of the drug with relevant continuous complete remission rates and overall good tolerability (29). Interestingly, deletion of PTPN1 and PTPN2 phosphatases was related to the genesis of crizotinib resistance by upregulating SHP2 (30). Consistently, combined blockage of ALK and SHP2 potentiated the efficacy of crizotinib in ALCL cells (30). Similarly, combinations of crizotinib with CHOP chemotherapy, decitabine and trametinib, or with second-generation ALK inhibitors often completely suppressed the emergence of resistant cells and were more effective than single drugs in the long-term control of lymphoma cells expansion, by inducing deeper inhibition of oncogenic signaling and higher rates of apoptosis (31).

Second generation ALK inhibitors also appeared very interesting. Alectinib showed favorable clinical activity and was well tolerated in patients with ALK‐positive ALCL who had progressed on standard chemotherapy (32). In a phase II clinical trial it induced objective responses in 8/10 patients, with 6 complete remissions (32). The 1‐year progression‐free survival, event‐free survival, and overall survival rates were 58.3%, 70.0%, and 70.0%, respectively (32).

## 3 JAK/STAT signaling in PTCLs

The Janus kinase (JAK)/signal transducer and activator of transcription (STAT) pathway are critical for blood formation and immune response (33). It includes over 30 transmembrane proteins that recognize specific cytokines, many of which transmit antiapoptotic, proliferative, and differentiation signals (33). Several cancers, including blood malignancies, have been associated with the constitutive activation of STAT family members, which normally require JAK-mediated tyrosine phosphorylation for transcriptional activation.

JAK/STAT signaling plays a prominent role in adult T-cell leukemia and lymphoma because of the effects of HTLV1 lymphomagenesis (34), T-large granular leukemia (35), T-lymphoblastic leukemia/lymphoma (35), cutaneous lymphomas (36, 37), and various nodal and extranodal TCLS (38). Notably, STAT3 is the main downstream effector of ALK in ALCL (22).

As far as nodal PTCLs are concerned, gene expression analyses first provided evidence for activation of the JAK/STAT pathway and the downstream nuclear factor kB (NF-kB) (39, 40), which was further confirmed by immunohistochemical detection of nuclear-phosphorylated STAT proteins (38). It is noteworthy that experimental retroviral-insertion mutagenesis performed on oligoclonal mature T-cells demonstrated the transforming potential of JAK1 in this setting through JAK/STAT pathway activation (41).

Several studies have consistently demonstrated the presence of recurrent mutations targeting the JAK/STAT pathways in various PTCLs, including NK/T-cell, nodal, and intestinal forms (42–44). However, it should be noted that the frequency of such mutations is relatively low (below 10%) in nodal PTCLs, mostly affecting JAK1, STAT3, and CCR4. In contrast, evidence of pathway activation is supported by nuclear staining of p-STAT3, p-STAT5, and p-STAT6 in at least 25–30% of cases (38, 44). Therefore, other activation mechanisms have been investigated. Common STAT activation mechanisms in PTCLs include the overexpression of TKs rather than ALK, such as LCK (45) or PDGRs (38, 46), microenvironment-mediated cytokine expression and interleukin-2 receptor engagement (47), and MTMR2 overexpression (48).

Interestingly, the activation of JAK/STAT and downstream NF-kB might represent a suitable therapeutic target in PTCLs. A recent phase I/II study tested ruxolitinib in cutaneous and nodal T-cell lymphomas. Cases were divided into three different cohorts based on the presence of activating mutations in JAK1, JAK2, JAK3, STAT3, or STAT5B (cohort1) or evidence of STAT3 activation by IHC in the absence of mutations (cohort 2). Patients enrolled in cohort 3 lacked both activation mutations and detection of nuclear p-STAT3. The overall clinical benefit rate (CBR) was defined as the combination of complete response (CR), partial response (PR), and stable disease lasting at least six months. Among the PTCL patients (n = 45), CBR was 53%, 45%, and 13% in cohorts 1, 2, and 3, respectively (P = .02). This indicated that the pre-treatment selection of patients could be very useful for effective direct treatment. Eight patients had a CBR > 12 months. Notably, the expression of phosphorylated S6, a marker of PI3 kinase or mitogen-activated protein kinase activation, was associated with clinical responses (P = .05) (49).

## 4 Platelet-derived growth factor receptors in PTCLs

**Figure 3:**
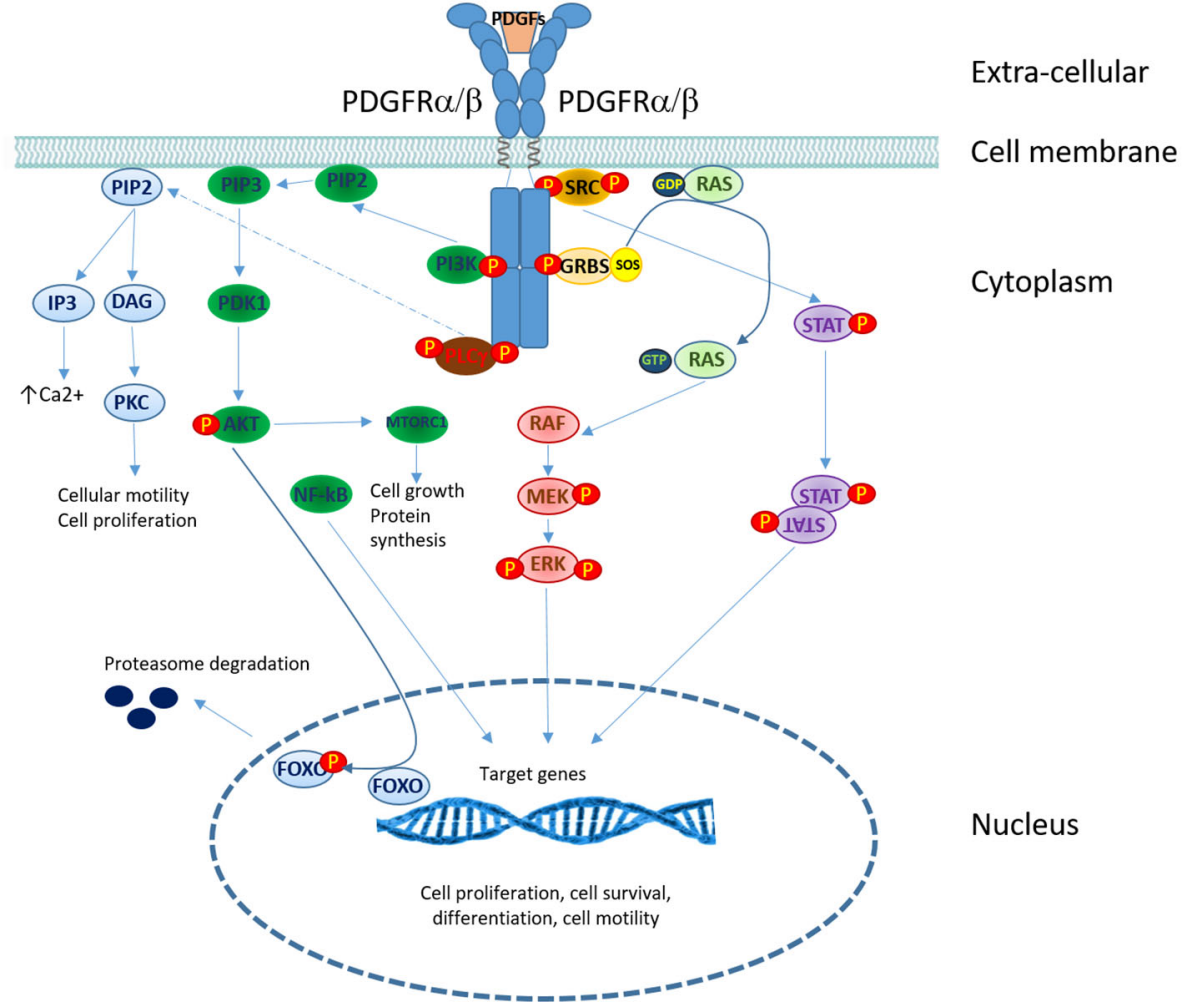
PDGFRs signaling in T-cell lymphomas. Overview of signaling pathways downstream of PDGF receptors. PDGF binding induces receptor dimerization and trans-phosphorylation. The recruitment of signaling and adaptor molecules to the phosphorylated receptor triggers several signaling cascades: phospholipase C γ (PLCγ), phosphatidylinositol-3-kinase (PI3K), mitogen-activated protein (MAP) kinases and signal transducers and activators of transcription (STATs). (P, phosphorylation site).

Platelet-derived growth factor (PDGF) receptors (PDGFRs) belong to the family of receptor tyrosine kinases (RTKs). There are two isoforms of PDGF receptors, PDGFRα and PDGFRβ, which are encoded by two different genes: *PDGFRA* and *PDGFRB*. Five different ligands (PDGF-AA, PDGF-BB, PDGF-AB, PDGF-CC, and PDGF-DD) can bind to receptors, inducing their dimerization and functional activation (50). Phospholipase Cγ (PLCγ), phosphatidylinositol- 3-kinase (PI3K), SRC family kinases, and STATs) are the main PDGFR signaling downstream components (46). MAP kinases and other adaptor molecules are also recruited to PDGFR, which regulates multiple pathways (50)(**Figure 3**).

PDGFRA was found to be consistently overexpressed in PTCL/NOS in terms of both mRNA and protein expression, based on gene expression profiling and tissue microarray analysis (51). Subsequently, PDGFRA activation was shown to be necessary for PTCL cell proliferation and survival, as exposure to imatinib mesylate led to cell cycle arrest and apoptosis *ex vivo* and *in vivo* (52). Interestingly, unlike other malignancies, PDGFRA activation is not due to somatic mutations or other genetic events. Rather, it has been shown that autocrine stimulation through PDGF ligands could activate the receptor *ex vivo* and *in vivo* (38). Without ligand availability, PTCL cells showed PDGFRA dephosphorylation and consistent inactivation of downstream STATs (38). PDGFRA expression was also been detected in TFH-related PTCL angioimmunoblastic type (AITL) (53), cutaneous lymphomas, and anaplastic large cell lymphoma (38). Consistent with these findings, an *in vivo* study was conducted to demonstrate the relevance of PDGFRs activation in maintaining PTCL cell vitality (54). Simultaneously, different studies have provided clinical evidence of the potential effectiveness of TKI in PTCLs (38, 54).

Based on this data, clinical trials have been conducted to test different TKIs. Dasatinib, a multi-target TKI, was shown to increase the efficacy of chemotherapy, particularly the CHOEP scheme, both *in vitro* and *in vivo* (55). When dasatinib was used in the relapsed/refractory setting, the overall response rate among 10 PTCL patients was approximately 50%; however, 2 patients with PTCL/NOS carrying *LRRK2* mutations achieved stable and durable complete remission (56). Despite the limited number of patients, sorafenib showed intriguing efficacy, inducing CR in 3/3 PTCL patients (2 AITL) (57).

## 5 Targeting TK downstream the T-cell receptor

The engagement of immunoreceptors results in a bright proliferative and metabolic response in T-cells. This response is largely mediated by the NF-kB signaling pathway (40). Indeed, in T-cell lymphomas, ITK and GATA3 are involved, and not only does the T-cell receptor (TCR) supports proliferation and survival but also directly maintains chemotherapy resistance (58). Several TK participating in the TCR complex or mediating downstream signals have been reported to be either activated by somatic mutations and/or chromosomal translocations or simply overexpressed in PTCLs.

The most commonly affected lesion appears to be TK SYK. Feldman et al. reported in 2008 that SYK was consistently overexpressed in PTCL (59), and SYK inhibition led to PTCL cell apoptosis in an experimental model (60). However, since then, the actual role of wild-type SYK in PTCLs has not been demonstrated. In contrast, SYK is involved in the most common translocation observed in TFH-related PTCLs, specifically in the follicular variant, t (5, 9)(q33;q22), leading to the fusion gene *ITK-SYK* (61). The ITK-SYK fusion protein acts as a constitutively active SYK tyrosine kinase with oncogenic properties *in vitro* and *in vivo* by mimicking a constitutively active TCR signal. The conditional expression of ITK/SYK recapitulated the development of PTCL in a mouse model for the first time *in vivo* (62). Further evidence has shown that the transforming potential of t (5, 9)(q33;q22) is mediated by IL2RG/JAK3/STA3-STAT5 activation and is associated with CD69 expression (63, 64). Notably, although AITL can present with ITK gains (65), ITK/SYK fusion is highly specific to follicular PTCL and is only exceptionally observed in AITL (66). Unfortunately, despite the rationale, the clinical use of ibrutinib, a Bruton tyrosine kinase inhibitor effective on ITK signaling, showed limited efficacy in PTCL in a pivotal trial (67).

Another TK associated with the TCR complex, which can be involved in chromosomal translocations, is FYN. FYN-TRAF3IP2 rearrangement was recently described in PTCL/NOS and is associated with NF-κB activation (68, 69). More rarely, LCK can hijack TCR signaling through KHDRBS1-LCK rearrangement (69).

## 6 Other tyrosine kinases in PTCLs

A few other TKs may have a relevant role in PTCL pathogenesis and may be good candidates as therapeutic targets.

Protein kinase C (PKC) is critical for T lymphocyte activation and proliferation. Different isoforms, including zeta, theta, and iota, are overexpressed in PTCLs (52, 70). They may promote cell survival through higher nitric oxide synthase (NOS) activity, which may represent a suitable target (71). Recently, Debackere et al. described t (1, 5)(p34;q21.3) and t (15, 16)(q26.1;q22.1) with ITK-FER and RLTPR-FES rearrangements, both acting through STAT3 phosphorylation (72).

Vascular endothelial growth factor receptor (VEGFR) is consistently overexpressed in AITL (53). Autocrine stimulation has been proposed as not being affected by genetic lesions of any type but being co-expressed with VEGF by neoplastic cells (1, 52, 53). However, no formal demonstration of this has been provided.

KIT is weakly expressed in approximately 30% of PTCL/NOS cases, presenting somatic mutations in exon 11 in only 5% of cases (72). Finally, B-lymphoid tyrosine kinase (BLK) expression in nodal PTCL should be evaluated, as it is commonly found in CTCL, and dasatinib showed some degree of effectiveness in such instances (73).

## 7 Conclusions and perspectives

Tyrosine kinases are often involved in the molecular pathogenesis of PTCL and are either activated by genetic events (somatic mutations and translocations) or simply overexpressed and activated by receptor engagement. Therefore, despite the existence of autocrine stimulation, the microenvironment plays a significant role.

So far, with few exceptions, TKIs have not yet shown prominent clinical activity. However, the strong biological rationale and the remarkable (though still anecdotic) experience in a few patients (Piccaluga (1, 52, 53); laimer; Umakanthan; gibson) suggest that this strategy should be better investigated. Furthermore, since TK signaling activates multiple downstream molecules, such as PI3K/AKT, JAK/STAT3-STAT5, mTOR, and SRC, it is reasonable to speculate that several small molecules targeting key effectors within these pathways should be investigated in PTCL patients. Additionally, given the enormous redundancy of signal transduction pathways in a given PTCL case, it is conceivable that using disease/patient-specific cocktails will be more effective in successfully knocking down multiple players among different pathways. Therefore, targeting STAT, EGFR, SRC, and MEK may also be considered. Finally, novel immunological strategies, including checkpoint inhibitors and epigenetic modifiers combined with TKI and chemotherapy, might be considered to enhance anti-tumor responses and eventually achieve complete eradication of lymphomatous cells.

## Author contributions

All authors listed have made a substantial, direct, and intellectual contribution to the work, and approved it for publication.

## References

[1] Piccaluga PP Paolini S Martinelli G. Tyrosine kinase inhibitors for the treatment of Philadelphia chromosome-positive adult acute lymphoblastic leukemia. Cancer. (2007) 110(6):1178–86. doi: 10.1002/cncr.22881 17701954

[2] Saraon P Pathmanathan S Snider J Lyakisheva A Wong V Stagljar I. Receptor tyrosine kinases and cancer: oncogenic mechanisms and therapeutic approaches. Oncogene. (2021) 40(24):4079–93. doi: 10.1038/s41388-021-01841-2 34079087

[3] Jiao Q Bi L Ren Y Song S Wang Q Wang YS. Advances in studies of tyrosine kinase inhibitors and their acquired resistance. Mol Cancer. (2018) 17(1):36. doi: 10.1186/s12943-018-0801-5 29455664 PMC5817861

[4] Huang L Jiang S Shi Y. Tyrosine kinase inhibitors for solid tumors in the past 20 years (2001-2020). J Hematol Oncol (2020) 13(1):143. doi: 10.1186/s13045-020-00977-0 33109256 PMC7590700

[5] Esteban-Villarrubia J Soto-Castillo JJ Pozas J Román-Gil MS Orejana-Martín I Torres-Jiménez J. Tyrosine kinase receptors in oncology. Int J Mol Sci (2020) 21(22):8529. doi: 10.3390/ijms21228529 33198314 PMC7696731

[6] Shimada A. Hematological malignancies and molecular targeting therapy. Eur J Pharmacol (2019) 862:172641. doi: 10.1016/j.ejphar.2019.172641 31493406

[7] Hou JZ Ye JC Pu JJ Liu H Ding W Zheng H. Novel agents and regimens for hematological malignancies: recent updates from 2020 ASH annual meeting. J Hematol Oncol (2021) 14(1):66. doi: 10.1186/s13045-021-01077-3 33879198 PMC8059303

[8] Burger JA. Bruton tyrosine kinase inhibitors: Present and future. Cancer J (2019) 25(6):386–93. doi: 10.1097/PPO.0000000000000412 PMC7083517 31764119

[9] Pal Singh S Dammeijer F Hendriks RW. Role of bruton's tyrosine kinase in b cells and malignancies. Mol Cancer. (2018) 17(1):57. Erratum in: Mol Cancer. 2019 Apr 3;18(1):79. doi: 10.1186/s12943-018-0779-z 29455639 PMC5817726

[10] Barreca A Lasorsa E Riera L Machiorlatti R Piva R Ponzoni M. Anaplastic lymphoma kinase in human cancer. J Mol Endocrinol (2011) 47(1):R11–23. doi: 10.1530/JME-11-0004 21502284

[11] Khoury JD Solary E Abla O Akkari Y Alaggio R Apperley JF. The 5th edition of the world health organization classification of haematolymphoid tumours: Myeloid and Histiocytic/Dendritic neoplasms. Leukemia (2022) 36:1703–19. doi: 10.1038/s41375-022-01613-1 PMC9252913 35732831

[12] Morris SW Kirstein MN Valentine MB Dittmer KG Shapiro DN Saltman DL. Fusion of a kinase gene, ALK, to a nucleolar protein gene, NPM, in non-hodgkin’s lymphoma. Science. (1994) 263:1281–4. doi: 10.1126/science.8122112 8122112

[13] Shiota M Fujimoto J Semba T Satoh H Yamamoto T Mori S. Hyperphosphorylation of a novel 80 kDa protein tyrosine kinase similar to ltk in a human ki-1 lymphoma cell line, AMS3. Oncogene. (1994) 9(6):1567–74. 8183550

[14] Webb TR Slavish J George RE Look AT Xue L Jiang Q. Anaplastic lymphoma kinase: role in cancer pathogenesis and small-molecule inhibitor development for therapy. Expert Rev Anticancer Ther (2009) 9(3):331–56. doi: 10.1586/14737140.9.3.331 PMC2780428 19275511

[15] Iwahara T Fujimoto J Wen D Cupples R Bucay N Arakawa T. Molecular characterization of ALK, a receptor tyrosine kinase expressed specifically in the nervous system. Oncogene (1997) 14:439–49. doi: 10.1038/sj.onc.1200849 9053841

[16] Mourali J Bénard A Lourenço FC Monnet C Greenland C Moog-Lutz. Anaplastic lymphoma kinase is a dependence receptor whose proapoptotic functions are activated by caspase cleavage. Mol Cell Biol (2006) 26:6209–22. doi: 10.1128/MCB.01515-05 PMC1592804 16880530

[17] Chiarle R Voena C Ambrogio C Piva R Inghirami G. The anaplastic lymphoma kinase in the pathogenesis of cancer. Nat Rev Cancer (2008) 8:11–23. doi: 10.1038/nrc2291 18097461

[18] Fornari A Piva R Chiarle R Novero D Inghirami G. Anaplastic large cell lymphoma: one or more entities among T-cell lymphoma? Hematological Oncol (2009) 27:161–70. doi: 10.1002/hon.897 19358142

[19] Okuwaki M. The structure and functions of NPM1/nucleophosmin/B23, a multifunctional nucleolar acidic protein. J Biochem (2008) 143:441–8. doi: 10.1093/jb/mvm222 18024471

[20] Fujimoto J Shiota M Iwahara T Seki N Satoh H Mori S. Characterization of the transforming activity of p80, a hyperphosphorylated protein in a ki-1 lymphoma cell line with chromosomal translocation t(2;5). PNAS (1996) 93:4181–6. doi: 10.1073/pnas.93.9.4181 PMC39508 8633037

[21] Ischof D Pulford K Mason DY Morris SW. Role of the nucleophosmin (NPM) portion of the non-hodgkin's lymphoma-associated NPM–anaplastic lymphoma kinase fusion protein in oncogenesis. Mol Cell Biol (1997) 17:2312–25. doi: 10.1128/MCB.17.4.2312 PMC232080 9121481

[22] Chiarle R Simmons WJ Cai H Dhall G Zamo A Raz R. Stat3 is required for ALK-mediated lymphomagenesis and provides a possible therapeutic target. Nat Med (2005) 11:623–9. doi: 10.1038/nm1249 15895073

[23] Piva R Chiarle R Manazza AD Taulli R Simmons W Ambrogio C. Ablation of oncogenic ALK is a viable therapeutic approach for anaplastic large-cell lymphomas. Blood (2006) 107:689–97. doi: 10.1182/blood-2005-05-2125 PMC1895619 16189272

[24] Gambacorti-Passerini C Messa C Pogliani EM. Crizotinib in anaplastic large-cell lymphoma. N Engl J Med (2011) 364(8):775–6. doi: 10.1056/NEJMc1013224 21345110

[25] Passerini CG Farina F Stasia A Redaelli S Ceccon M Mologni L. Crizotinib in advanced, chemoresistant anaplastic lymphoma kinase-positive lymphoma patients. J Natl Cancer Inst (2014) 106(2):djt378. doi: 10.1093/jnci/djt378 24491302

[26] Gambacorti-Passerini C Orlov S Zhang L Braiteh F Huang H Esaki T. Long-term effects of crizotinib in ALK-positive tumors (excluding NSCLC): A phase 1b open-label study. Am J Hematol (2018) 93(5):607–14. doi: 10.1002/ajh.25043 PMC5947833 29352732

[27] Bossi E Aroldi A Brioschi FA Steidl C Baretta S Renso R. Phase two study of crizotinib in patients with anaplastic lymphoma kinase (ALK)-positive anaplastic large cell lymphoma relapsed/refractory to chemotherapy. Am J Hematol (2020) 95(12):E319–21. doi: 10.1002/ajh.25967 32808682

[28] Merino M Kasamon Y Li H Ma L Leong R Zhou J. FDA Approval summary: Crizotinib for pediatric and young adult patients with relapsed or refractory systemic anaplastic large cell lymphoma. Pediatr Blood Cancer. (2022) 69(8):e29602. doi: 10.1002/pbc.29602 35561013

[29] Rindone G Aroldi A Bossi E Verga L Zambrotta G Tarantino S. A monocentric analysis of the long-term safety and efficacy of crizotinib in relapsed/refractory ALK+ lymphomas. Blood Adv (2023) 7(3):314–6. doi: 10.1182/bloodadvances.2022007538 PMC9898594 35914224

[30] Karaca Atabay E Mecca C Wang Q Ambrogio C Mota I Prokoph N. Tyrosine phosphatases regulate resistance to ALK inhibitors in ALK+ anaplastic large cell lymphoma. Blood. (2022) 139(5):717–31. doi: 10.1182/blood.2020008136 PMC8814675 34657149

[31] Arosio G Sharma GG Villa M Mauri M Crespiatico I Fontana D. Synergistic drug combinations prevent resistance in ALK+ anaplastic Large cell lymphoma. Cancers (Basel). (2021) 13(17):4422. doi: 10.3390/cancers13174422 34503232 PMC8431561

[32] Fukano R Mori T Sekimizu M Choi I Kada A Saito AM. Alectinib for relapsed or refractory anaplastic lymphoma kinase-positive anaplastic large cell lymphoma: An open-label phase II trial. Cancer Sci (2020) 111(12):4540–7. doi: 10.1111/cas.14671 PMC7734006 33010107

[33] Vainchenker W Constantinescu SN. JAK/STAT signaling in hematological malignancies. Oncogene. (2013) 32(21):2601–13. doi: 10.1038/onc.2012.347 22869151

[34] Waldmann TA Chen J. Disorders of the JAK/STAT pathway in T cell lymphoma pathogenesis: Implications for immunotherapy. Annu Rev Immunol (2017) 35:533–50. doi: 10.1146/annurev-immunol-110416-120628 PMC7974381 28182501

[35] Teramo A Barilà G Calabretto G Ercolin C Lamy T Moignet A. *STAT3* mutation impacts biological and clinical features of T-LGL leukemia. Oncotarget. (2017) 8(37):61876–89. doi: 10.18632/oncotarget.18711 PMC5617471 28977911

[36] Pérez C González-Rincón J Onaindia A Almaráz C García-Díaz N Pisonero H. Mutated JAK kinases and deregulated STAT activity are potential therapeutic targets in cutaneous T-cell lymphoma. Haematologica. (2015) 100(11):e450–3. doi: 10.3324/haematol.2015.132837 PMC4825308 26294736

[37] Lee K Evans MG Yang L Ng S Snowden C Khodadoust M. Primary cytotoxic T-cell lymphomas harbor recurrent targetable alterations in the JAK-STAT pathway. Blood. (2021) 138(23):2435–40. doi: 10.1182/blood.2021012536 PMC8662071 34432866

[38] Piccaluga PP Rossi M Agostinelli C Carbone A Fantoni L Ferrari S. Platelet-derived growth factor alpha mediates the proliferation of peripheral T-cell lymphoma cells *via* an autocrine regulatory pathway. Leukemia. (2014) 28(8):1687–97. doi: 10.1038/leu.2014.50 24480986

[39] Tang T Allen G Koo GC Tay K Tan D Quek R. Gene expression profiling identifies the JAK/STAT and NFκB pathways to be important in peripheral T-cell lymphomas and natural-killer T-cell lymphomas. Blood (2011) 118(21):2658. doi: 10.1182/blood.v118.21.2658.2658

[40] Navari M Etebari M Ricci F. T-Cell receptor dependent and independent NF-kappa b activation is a prognostic marker and a therapeutic target in peripheral T-cell lymphoma not otherwise specified. precision medicine in emergency medicine. Digit. Med Health Technol (2022) 1. doi: 10.5772/dmht.04

[41] Heinrich T Rengstl B Muik A Petkova M Schmid F Wistinghausen R. Mature T-cell lymphomagenesis induced by retroviral insertional activation of janus kinase 1. Mol Ther (2013) 21(6):1160–8. doi: 10.1038/mt.2013.67 PMC3677306 23609016

[42] Koo GC Tan SY Tang T Poon SL Allen GE Tan L. Janus kinase 3-activating mutations identified in natural killer/T-cell lymphoma. Cancer Discovery (2012) 2(7):591–7. doi: 10.1158/2159-8290.CD-12-0028 22705984

[43] Nicolae A Xi L Pham TH Pham TA Navarro W Meeker HG. Mutations in the JAK/STAT and RAS signaling pathways are common in intestinal T-cell lymphomas. Leukemia. (2016) 30(11):2245–7. doi: 10.1038/leu.2016.178 PMC5093023 27389054

[44] Manso R Sánchez-Beato M González-Rincón J. Mutations in the JAK/STAT pathway genes and activation of the pathway, a relevant finding in nodal peripheral T-cell lymphoma. Br J Haematol (2018) 183(3):497–501. doi: 10.1111/bjh.14984 29076126

[45] Yu CL Jove R Burakoff SJ. Constitutive activation of the janus kinase-STAT pathway in T lymphoma overexpressing the lck protein tyrosine kinase. J Immunol (1997) 159(11):5206–10. doi: 10.4049/jimmunol.159.11.5206 9548458

[46] Pileri SA Piccaluga PP. New molecular insights into peripheral T cell lymphomas. J Clin Invest. (2012) 122(10):3448–55. doi: 10.1172/JCI61205 PMC3461903 23023716

[47] Zhang Q Nowak I Vonderheid EC Rook AH Kadin ME Nowell PC. Activation of Jak/STAT proteins involved in signal transduction pathway mediated by receptor for interleukin 2 in malignant T lymphocytes derived from cutaneous anaplastic large T-cell lymphoma and sezary syndrome. Proc Natl Acad Sci U S A. (1996) 93(17):9148–53. doi: 10.1073/pnas.93.17.9148 PMC38610 8799169

[48] Wang J He AL Zhang WG Cao XM Chen YX Liu J. MTMR2 promotes the progression of NK/T cell lymphoma by targeting JAK1. Eur Rev Med Pharmacol Sci (2020) 24(15):8057–66. doi: 10.1158/1078-0432.CCR-16-1996 32767332

[49] Moskowitz AJ Ghione P Jacobsen E Ruan J Schatz JH Noor S. A phase 2 biomarker-driven study of ruxolitinib demonstrates effectiveness of JAK/STAT targeting in T-cell lymphomas. Blood. (2021) 138(26):2828–37. doi: 10.1182/blood.2021013379 PMC8718625 34653242

[50] Guérit E Arts F Dachy G Boulouadnine B Demoulin JB. PDGF receptor mutations in human diseases. Cell Mol Life Sci (2021) 78(8):3867–81. doi: 10.1007/s00018-020-03753-y PMC11072557 33449152

[51] Piccaluga PP Agostinelli C Zinzani PL Rossi M Basso K Zupo S. Expression of platelet-derived growth factor receptor alpha in peripheral T-cell lymphoma not otherwise specified. Lancet Oncol (2005) 6(6):440. doi: 10.1016/S1470-2045(05)70213-8 15925824

[52] Piccaluga PP Agostinelli C Califano A Carbone A Fantoni L Ferrari S. Gene expression analysis of peripheral T cell lymphoma, unspecified, reveals distinct profiles and new potential therapeutic targets. J Clin Invest. (2007) 117(3):823–34. doi: 10.1172/JCI26833 PMC1794115 17304354

[53] Piccaluga PP Agostinelli C Califano A Baccarani M Favera RD Pileri SA. Gene expression analysis of angioimmunoblastic lymphoma indicates derivation from T follicular helper cells and vascular endothelial growth factor deregulation. Cancer Res (2007) 67(22):10703–10. doi: 10.1158/0008-5472.CAN-07-1708 18006812

[54] Laimer D Dolznig H Kollmann K Vesely PW Schlederer M Merkel O. PDGFR blockade is a rational and effective therapy for NPM-ALK-driven lymphomas. Nat Med (2012) 18(11):1699–704. doi: 10.1038/nm.2966 23064464

[55] Magni M Biancon G Rizzitano S Cavanè A Paolizzi C Dugo M. Tyrosine kinase inhibition to improve anthracycline-based chemotherapy efficacy in T-cell lymphoma. Br J Cancer. (2019) 121(7):567–77. doi: 10.1038/s41416-019-0557-8 PMC6889385 31474759

[56] Umakanthan JM Iqbal J Batlevi CL Bouska A Smith LM Shostrom V. Phase I/II study of dasatinib and exploratory genomic analysis in relapsed or refractory non-Hodgkin lymphoma. Br J Haematol (2019) 184(5):744–52. doi: 10.1111/bjh.15702 PMC8281362 30520026

[57] Gibson JF Foss F Cooper D Seropian S Irizarry D Barbarotta L. Pilot study of sorafenib in relapsed or refractory peripheral and cutaneous T-cell lymphoma. Br J Haematol (2014) 167(1):141–4. doi: 10.1111/bjh.12944 24888971

[58] Wang T Lu Y Polk A Zamalloa CM Fujiwara H Suemori K. T-Cell receptor signaling activates an ITK/NF-κB/GATA-3 axis in T-cell lymphomas facilitating resistance to chemotherapy. Clin Cancer Res (2017) 23(10):2506–15. doi: 10.1158/1078-0432.CCR-16-1996 PMC5405012 27780854

[59] Feldman AL Sun DX Law ME Novak AJ Attygalle AD Thorland EC. Overexpression of syk tyrosine kinase in peripheral T-cell lymphomas. Leukemia. (2008) 22(6):1139–43. doi: 10.1038/leu.2008.77 PMC2778211 18401419

[60] Wilcox RA Sun DX Novak A Dogan A Ansell SM Feldman AL. Inhibition of syk protein tyrosine kinase induces apoptosis and blocks proliferation in T-cell non-hodgkin's lymphoma cell lines. Leukemia. (2010) 24(1):229–32. doi: 10.1038/leu.2009.198 PMC3122132 19776763

[61] Streubel B Vinatzer U Willheim M Raderer M Chott A. Novel t(5;9)(q33;q22) fuses ITK to SYK in unspecified peripheral T-cell lymphoma. Leukemia. (2006) 20(2):313–8. doi: 10.1038/sj.leu.2404045 16341044

[62] Pechloff K Holch J Ferch U. The fusion kinase ITK-SYK mimics a T cell receptor signal and drives oncogenesis in conditional mouse models of peripheral T cell lymphoma. J Exp Med (2010) 207(5):1031–44. doi: 10.1084/jem.20092042 PMC2867290 20439541

[63] Fathi NN Mohammad DK Görgens A Schweneker M Brunner K Kremer M. Translocation-generated ITK-FER and ITK-SYK fusions induce STAT3 phosphorylation and CD69 expression. Biochem Biophys Res Commun (2018) 504(4):749–52. doi: 10.1016/j.bbrc.2018.09.019 30217447

[64] Zhang LL Pan HX Wang YX Guo T Liu L. Genome profiling revealed the activation of IL2RG/JAK3/STAT5 in peripheral T cell lymphoma expressing the ITK SYK fusion gene. Int J Oncol (2019) 55(5):1077–89. doi: 10.3892/ijo.2019.4882 PMC6776186 31545408

[65] Liang PI Chang ST Lin MY Hsieh YC Chu PY Chen CJ. Angioimmunoblastic T-cell lymphoma in Taiwan shows a frequent gain of ITK gene. Int J Clin Exp Pathol (2014) 7(9):6097–107. doi: 10.1097/01.pat.0000454436.01801.25 PMC4203228 25337257

[66] Attygalle AD Feldman AL Dogan A. ITK/SYK translocation in angioimmunoblastic T-cell lymphoma. Am J Surg Pathol (2013) 37(9):1456–7. doi: 10.1097/PAS.0b013e3182991415 24076779

[67] Kumar A Vardhana S Moskowitz AJ Porcu P Dogan A Dubovsky JA. Pilot trial of ibrutinib in patients with relapsed or refractory T-cell lymphoma. Blood Adv (2018) 2(8):871–6. doi: 10.1182/bloodadvances.2017011916 PMC5915998 29669753

[68] Moon CS Reglero C Cortes JR Quinn SA Alvarez S Zhao J. FYN-TRAF3IP2 induces NF-κB signaling-driven peripheral T cell lymphoma. Nat Cancer. (2021) 2(1):98–113. doi: 10.1038/s43018-020-00161-w 33928261 PMC8081346

[69] Debackere K Marcelis L Demeyer S Ferreiro JAF van Roosbroeck K Marcelis L. Fusion transcripts FYN-TRAF3IP2 and KHDRBS1-LCK hijack T cell receptor signaling in peripheral T-cell lymphoma, not otherwise specified. Nat Commun (2021) 12(1):3705. doi: 10.1038/s41467-021-24037-4 34140493 PMC8211700

[70] Gorelik G Barreiro Arcos ML Klecha AJ Cremaschi GA. Differential expression of protein kinase c isoenzymes related to high nitric oxide synthase activity in a T lymphoma cell line. Biochim Biophys Acta (2002) 1588(2):179–88. doi: 10.1016/S0925-4439(02)00163-1 12385783

[71] Debackere K van der Krogt JA Tousseyn T Ferreiro JAF van Roosbroeck K Marcelis L. FER and FES tyrosine kinase fusions in follicular T-cell lymphoma. Blood. (2020) 135(8):584–8. doi: 10.1182/blood.2019002401 31746983

[72] Choe YS Kim JG Sohn SK Kim DH Baek JH Lee KB. C-kit expression and mutations in peripheral T cell lymphomas, except for extra-nodal NK/T cell lymphomas. Leuk Lymphoma. (2006) 47(2):267–70. doi: 10.1080/10428190500281680 16321856

[73] Petersen DL Krejsgaard T Berthelsen J Fredholm S Willerslev-Olsen A Sibbesen NA. B-lymphoid tyrosine kinase (Blk) is an oncogene and a potential target for therapy with dasatinib in cutaneous T-cell lymphoma (CTCL). Leukemia. (2014) 28(10):2109–12. doi: 10.1038/leu.2014.192 PMC4190403 24919804

